# Treatment and Characteristics of Pachychoroid Neovasculopathy Accompanying Recalcitrant Intraretinal Cysts

**DOI:** 10.7759/cureus.68174

**Published:** 2024-08-30

**Authors:** Moon Young Choi, Won Ki Lee

**Affiliations:** 1 Retina Center, Nune Eye Hospital, Seoul, KOR

**Keywords:** aflibercept intravitreal injection, intravitreal bevacizumab, intravitreal injections, amd, age-related macular degeneration (armd), pachychoroid, pachychoroid neovasculopathy, intraretinal fluid, anti-vegf vitreous injection

## Abstract

Purpose: To evaluate the clinical characteristics associated with chronic pachychoroid neovasculopathy (PNV) accompanying recalcitrant intraretinal cysts.

Methods: This is a retrospective, single-center, case-series study involving 20 eyes of 18 patients with PNV who did not respond to bevacizumab or ranibizumab and had to switch to aflibercept. Optical coherence tomography images were assessed before and after switching of intravitreal injection drug.

Results: The intraretinal cysts involved the outer nuclear layer and inner nuclear layer in 15 patients (75.0%), and involved only the inner nuclear layer in five patients (25.0%). All participants showed retinal pigment epithelium atrophy and outer retinal layer defect including external limiting membrane defect co-localized to the intraretinal cystic lesion. With the initial injection, bevacizumab and ranibizumab injections did not show a significant decrease in choroidal thickness (CT). Twenty (100.0%) patients showed poor response of intraretinal cyst response (IRCR). After a switch to aflibercept, IRCR was good in 17 (85.0%) patients and poor in three (15.0%) patients. Reduction of CT was great in aflibercept injections (from 229.0 μm to 204.0 μm, median, p < 0.001). Best-corrected visual acuity did not show significant improvement before or after switching drugs.

Conclusion: Intravitreal aflibercept injections were more effective in the reduction of CT and IRCR than bevacizumab and ranibizumab injections. The primary source of the intraretinal cyst fluid could be from the choroid.

## Introduction

Pachychoroid spectrum disease (PSD) is a group of disorders that share characteristics of thicker choroid, although not always, attenuation of choriocapillaries by underlying dilated Haller vessels, and choroidal vascular hyperpermeability [1]. Dilated Haller vessels, also known as pachyvessels, are thought to be caused by hydrostatic forces by an overloaded venous outflow system thus causing choroidal hyperpermeability. 

One of PSD is peripapillary pachychoroid syndrome. Peripapillary pachychoroid syndrome is a term described by Phasukkijwatana et al. in 2018 as a PSD variant characterized by a thickened nasal macular choroid with associated intraretinal and subretinal fluids in the nasal macular region extending from the disc margin [2]. Since the location is away from the macula, the symptomless nature of the disease often leads to chronic cases. Intraretinal fluid is usually a characteristic of the chronic stage. All of the eyes in the study had atrophy of the retinal pigment epithelium (RPE), ellipsoid zone, and external limiting membrane (ELM) at the peripapillary area suggesting the possibility of choroidal fluid entry into the retina, explaining the intraretinal fluid [2]. 

Pachychoroid neovasculopathy (PNV), another member of PSD, shares the common characteristics of PSD and develops type 1 neovascularization after chronic RPE and choriocapillaries damage without the presence of typical signs of age-related macular degeneration, such as soft drusen. In optical coherence tomography (OCT), the presence of type 1 neovascularization appears as a shallow irregular separation between RPE and Bruch’s membrane, also known as the “double layer sign” [3]. This is observed overlying dilated Haller vessels and attenuated choriocapillaries. OCT-angiography has allowed us to identify a tangled vascular network of flow signals between RPE and Bruch’s membrane often reflecting the presence of macular neovascularization (MNV). In these settings, if subretinal fluid or intraretinal fluid is present, MNV is thought to be exudative. Especially, in chronic pachychoroid settings, intraretinal cysts are often observed [4]. A study reported PNV eyes with macular retinoschisis-like intraretinal cysts and revealed that all of the eyes had RPE atrophy and defects of the outer retinal layers above pachyvessels [5]. Baek et al. suggested that intra-retinal fluid in the subjects is thought to occur due to a deficit of RPE and outer retinal layers including ELM, which facilitates the entry of choroidal fluid into intra-retinal space [5].

Thus, in cases in which neovascularization and outer retinal defect co-exist, the origin of intraretinal fluid is ambiguous because it may not always be due to macular neovascular activity. The source of the fluid could be from either the neovascular lesion or a mechanism related to non-neovascular hyperpermeable choroid origin. The effectiveness of bevacizumab and ranibizumab in PNV patients seems to be variable which may be due to unclear sources of fluid either from MNV or choroid [6]. Non-response in bevacizumab or ranibizumab, in spite of continued injections, is often observed and necessitates a switch to an alternative treatment such as aflibercept [7]. The purpose of this study was to evaluate the clinical characteristics of chronic PNV patients who were recalcitrant to bevacizumab or ranibizumab and switched to aflibercept.

## Materials and methods

Study subjects 

This retrospective case series study was conducted at a single institution using data from May 2019 to June 2023. The inclusion criteria were (1) PNV patients who showed shallow irregular RPE elevation on OCT and showed a tangled network of flow signal on OCT-Angiography (PLEX Elite 9000, Carl Zeiss AC, Jena, Germany or Optovue RTVue XR, Optovue Inc., Fremont, USA); (2) patients who had a schisis-like intraretinal cyst on OCT; and (3) patients who showed no response to continued bevacizumab or ranibizumab injections and had to switch to aflibercept. The exclusion criteria were ocular surgery within six months, and any accompanying conditions that can cause a retinoschisis or cystoid macular edema (CME) like appearances (X-linked retinoschisis, uveitis, optic nerve defect, glaucoma, vitreomacular traction, epiretinal membrane, diabetic retinopathy, retinal vein occlusion), and any opacity of media in the eye that hindered image quality for evaluation.

Design

**Figure 1:**
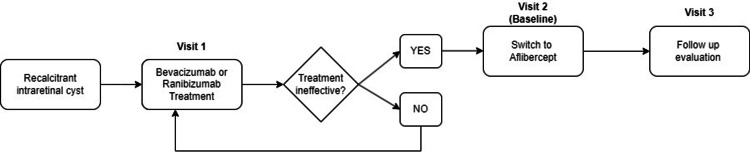
Study design. Visit 1 represents the last injection of either bevacizumab or ranibizumab. After one month, at visit 2, the practitioner decided to switch to aflibercept because the patient’s response to bevacizumab or ranibizumab was not sufficient. After one month from visit 2, at visit 3, the evaluation of the patient’s response to aflibercept was made by measuring choroidal thickness and intraretinal cyst response.

The measurements were made at a total of three visits. Visit 2 is the baseline point at which the practitioner decided that the response to bevacizumab or ranibizumab was not effective enough and had to switch to aflibercept for the first time. Visit 1 would be the last injection of bevacizumab or ranibizumab. Visit 3 was the first evaluation after switching to aflibercept. The intervals between the visits were always one month. The design is presented in Figure 1. 

The participants’ age, sex, ethnicity, and underlying disease history were taken at their first visit to the hospital. At all visits, Snellen best corrected visual acuity (BCVA), refractive error, widefield fundus photography (Optos, Optos PLC, Dunfermline, UK), and spectral domain OCT (Spectralis HRA + OCT; Heidelberg Engineering, Heidelberg, Germany) were taken. Some patients received fluorescein angiography (FA) and indocyanine green angiography (HRA2; Heidelberg Engineering, Heidelberg, Germany) during the course of follow-up, but not at visit 1. The type and date of intravitreal anti-vascular endothelial growth factor (VEGF) injections were recorded. Any other treatments including photodynamic therapy and focal laser were also recorded.

Imaging analysis

Choroidal thickness (CT) was measured manually using Heidelberg Eye Explorer software (Spectralis HRA-OCT; Heidelberg Engineering, Heidelberg, Germany) viewer at all visits. The vertical distance from Bruch’s membrane to the chorioscleral border was measured at 7 points, and the average of those 7 points was defined as the CT. These points were measured at 7 equidistant points along a horizontal line scan. These points divided the distance from the foveal center to the end of Bruch’s membrane nasally and temporally into four equal increments. The method was described in a previous work done on peripapillary polypoidal choroidal vasculopathy (PCV) patients [8]. Contrast and brightness were adjusted when needed to make precise measurements. 

Intraretinal cyst response (IRCR) was evaluated by observing the OCT at visit 2 and visit 3. “Good response” to anti-VEGF treatment was defined as complete resolution or near complete resolution of intraretinal fluid. Any status that did not match the definition of good response was categorized as “poor response.” Due to the difficulty of quantifying the volume of intraretinal fluid in the cases, binary categorization was chosen for the evaluation of responsiveness to different injections.

Statistical analysis

All data were first evaluated for normality using the Kolmogorov-Smirnov and Shapiro-Wilk tests. Given the non-parametric nature of our data, CT comparisons were performed using the Wilcoxon signed-rank test. BCVA was converted to a logarithm of the minimum angle of resolution (logMAR) for analysis. A p-value of less than 0.05 was considered statistically significant. All statistical analyses were conducted using IBM SPSS Statistics for Windows, Version 26, Released 2019 (IBM Corp., Armonk, NY).

Ethical considerations

This study was done in accordance with the Declaration of Helsinki. Due to the retrospective nature of this study, the Institutional Review Board/Ethics Committee ruled that approval was not required for this study.

## Results

**Figure 2:**
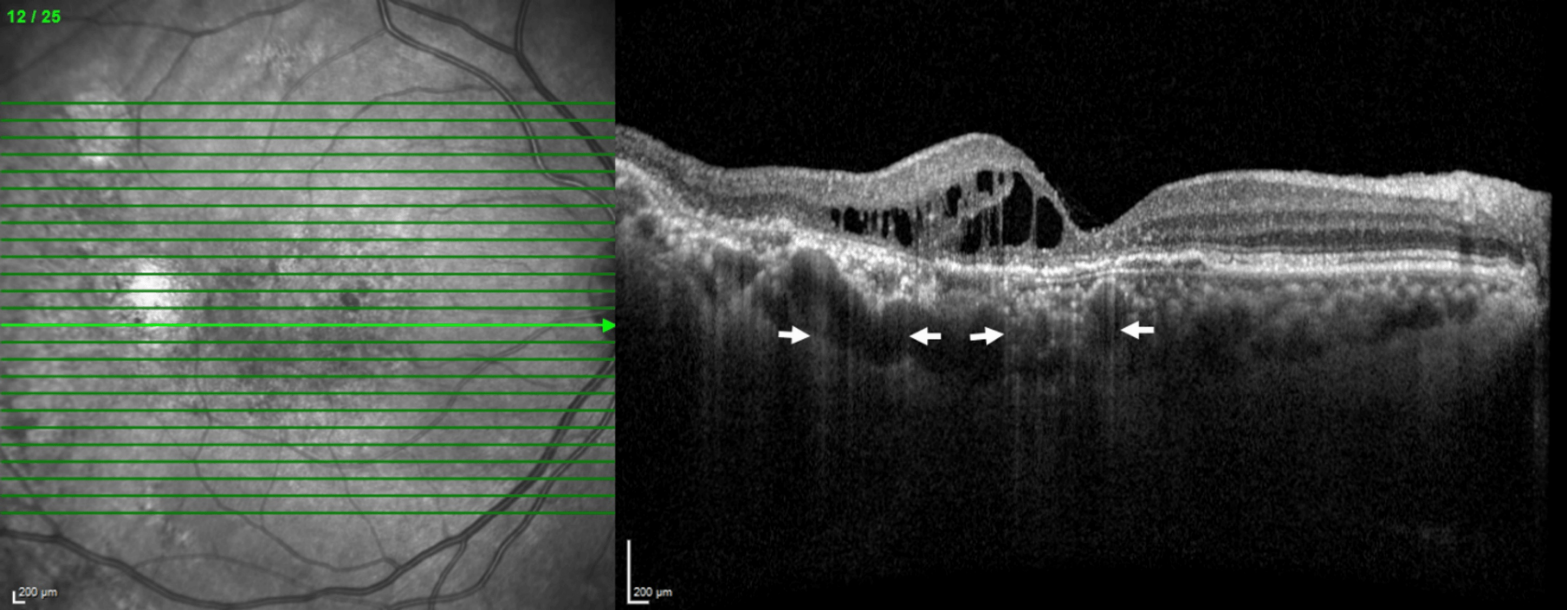
Optical coherence tomography of patient 3. Beneath the site of the intraretinal cyst, the arrows point to hyper-transmission into the choroid due to an RPE defect, and above that site, an ELM defect is observed. RPE, retinal pigment epithelium; ELM, external limiting membrane.

The analysis incorporated data from 20 eyes of 18 participants. The participants' average age was 71.22±9.67 years, spanning a range from 45 to 87 years, with 13 males (72.2%) and five females (27.85%). Retinoschisis-like intraretinal cysts were observed in the right eye in 50% of cases. There were no patients with central serous chorioretinopathy history. The extent of the schisis-like intraretinal cysts was found to involve both the outer nuclear layer and inner nuclear layer in 15 (75.0%) cases and only the outer nuclear layer in five cases (25.0%). All of the OCT results showed RPE atrophy which was confirmed by findings of RPE defects and hyper-transmission into the choroid at the location of the RPE defect. Typical ELM defects were also observed as the site of the RPE defects shown in Figure 2.

**Figure 3:**
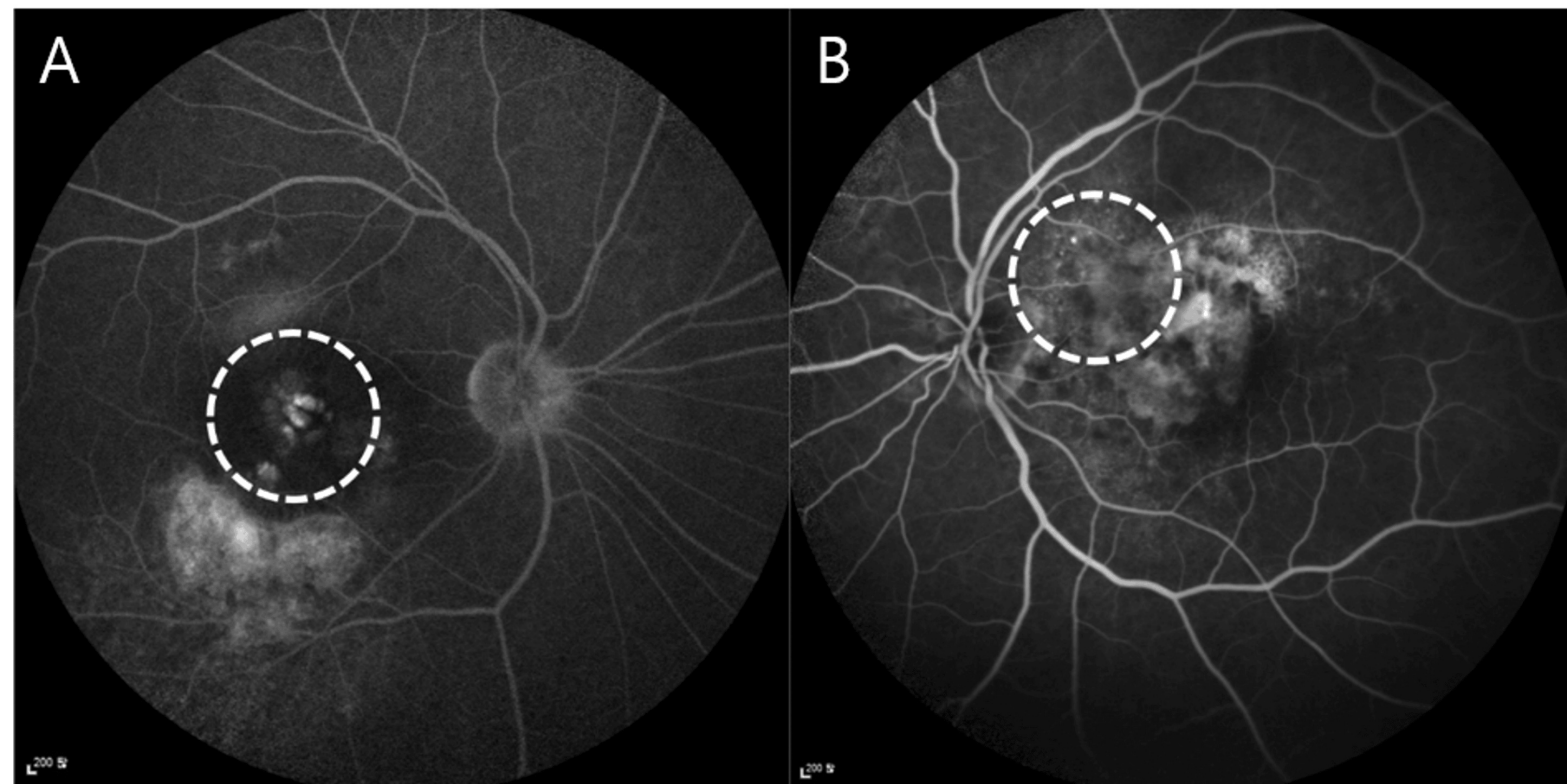
Fluorescein angiography of patient 5 (A) and patient 8 (B). (A) A petaloid pattern is observed (dotted circle). (B) Only a limited leaky pattern is observed (dotted circle) at the location of schisis-like intraretinal cysts.

FA patterns were analyzed for leaks, and the petaloid pattern of classic CME was identified in only four cases (Figure 3A). The remaining cases showed only limited leaks (Figure 3B).

Pigment epithelial detachment was observed in six eyes, hyperreflective foci was observed in 16 eyes and choroidal vascular hyperpermeability on mid to late phase of indocyanine green angiography was observed in 12 of 13 eyes. The clinical characteristics of the eyes in the study are summarized in Table 1.

**Table 1:** Clinical characteristics of the 20 eyes in this study. No., number; IRC, intraretinal cyst; RPE, retinal pigment epithelium; ELM, external limiting membrane; FA, fluorescein angiography; PED, pigment epithelial detachment; B, bevacizumab; R, ranibizumab; A, aflibercept; ONL, outer nuclear layer; INL, inner nuclear layer; Y, yes; N, no.

Patient No.	Injection sequence before switching to aflibercept	Extent of IRC	RPE atrophy	ELM defect	Petaloid leak on FA	PED	Hyper-reflective foci	Choroidal hyperpermeability
1	R1,B2,R2->A	ONL, INL	Y	Y	N	N	Mild	Y
2	B3->A	ONL, INL	Y	Y	N	N	Mild	Y
3	B1->A	ONL, INL	Y	Y	N	N	Rare	Y
	B1->A	ONL, INL	Y	Y	Y	N	None	Y
4	B3->A	ONL, INL	Y	Y	-	N	None	-
5	B1->A	ONL	Y	Y	Y	Y	Mild	Y
6	B1,R1,B3,R1->A	ONL, INL	Y	Y	N	N	None	Y
7	B7->A	ONL, INL	Y	Y	N	N	Moderate	-
8	B4->A	ONL	Y	Y	N	N	None	-
9	B2->A	ONL	Y	Y	N	Y	Rare	-
10	B1->A	ONL, INL	Y	Y	N	Y	Moderate	N
	B2->A	ONL	Y	Y	N	Y	Mild	Y
11	B1R3B1->A	ONL, INL	Y	Y	N	N	Rare	Y
12	B8->A	ONL	Y	Y	Y	N	Moderate	Y
13	B1->A	ONL, INL	Y	Y	Y	N	Rare	-
14	A6B1->A	ONL, INL	Y	Y	N	Y	Moderate	Y
15	B3->A	ONL, INL	Y	Y	-	N	Severe	-
16	A3B3->A	ONL, INL	Y	Y	N	N	Rare	Y
17	A1B1A1B1A2B2->A	ONL, INL	Y	Y	N	Y	Mild	Y
18	B5->A	ONL, INL	Y	Y	N	N	Rare	-

Bevacizumab and aflibercept were administered to all 20 eyes at least once whereas ranibizumab was used in three eyes. At the baseline, all eyes had a schisis-like intraretinal cyst. None of the participants received topical eye drops such as carbonic anhydrase inhibitors or intravitreal steroids. Two patients were documented as having undergone photodynamic therapy previously, and one participant had received focal laser photocoagulation previously. 

The main outcome of our investigation was the comparison of CT and IRCR at visit 2 and visit 3. Of the 20 eyes, 18 eyes received bevacizumab as the pre-switch injection, and two eyes received ranibizumab. Visit 2 evaluation showed a slight increase in CT from a median of 228.79 µm (interquartile range 181.79, 274.46) to a median of 229.00 µm (interquartile range 183.07, 278.07) but did not demonstrate significant alteration (p=0.444). A significant decrease of CT at visit 3 was observed by decreasing from a median of 229.00 µm to a median of 204.00 µm (interquartile range 133.36, 243.93) (p<0.001). These values are presented in Table 2.

**Table 2:** Changes in subfoveal choroidal thickness before and after switching injections. CT, choroidal thickness. *p-value<0.05. The data are presented as median (interquartile range). The z- and p-values are derived from the Wilcoxon signed-rank test.

	Variable	Pre-treatment (µm)	Post-treatment (µm)	z	p-value
Pre-switch	CT	228.79 (181.79,274.46)	229.00 (183.07,278.07)	-0.765	0.444
Post-switch	CT	229.00 (183.07,278.07)	204.00 (133.36,243.93)	-3.696	<0.001*

**Figure 4:**
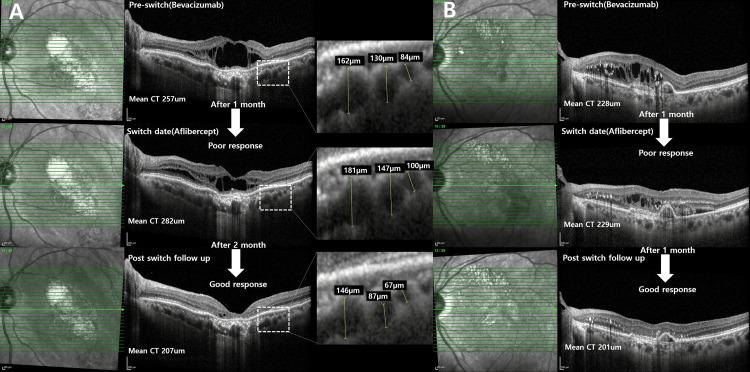
Optical coherence tomography of patient 4 (A) and patient 17 (B) before and after switch injection. A) After bevacizumab injection, there was poor response after one-month interval. The initial choroidal thickness was 257 µm. After switching to aflibercept, intraretinal cyst response showed good response and subfoveal choroidal thickness decreased from 282 µm to 207 µm at two months interval. The dotted rectangle was magnified four times to visualize the vertical diameter of Haller's vessel changes at pre-switch, switch date, and post-switch. After the switch to aflibercept, the diameter of Haller's vessel at the same location decreased compared to the two previous visits. (B) After bevacizumab injection, intraretinal cyst response showed poor response after a one-month interval and the mean choroidal thickness (CT) initially recorded as 228 µm, remained almost unchanged at the next follow-up. After the injection was switched to aflibercept, intraretinal cyst response was a good response and the mean CT decreased to 201 µm at the one-month follow-up.

IRCR was also evaluated, and at visit 2, there were 20 poor responses (100%), whereas at visit 3 there were three poor responses (15%) and 17 good responses (85%). Figures 4A, 4B display two representative patients with a decrease in CT and achieving complete resolution of intraretinal cysts at visit 3.

The secondary outcome was the comparison of BCVA before and after each injection. At visit 2, BCVA improved in six eyes (30%), remained the same in eight eyes (40%), and worsened in six eyes (30%). At visit 3, BCVA improved in seven eyes (35%), remained the same in nine eyes (45%), and worsened in four eyes (20%). Overall, BCVA at visit 1 was logarithm of the minimum angle of resolution (logMAR) 0.72 and at visit 2 was logMAR 0.7 (p=0.288). BCVA at visit 3 was logMAR 0.580 (p=0.861) which showed no statistically significant alterations associated with the two types of intravitreal injections (Table 3).

**Table 3:** Best corrected visual acuity (logMAR) following different types of injections. LogMAR, logarithm of the minimal angle of resolution. *p-value<0.05. The data are presented as median (interquartile range). The z- and p-values are derived from the Wilcoxon signed-rank test.

	Pre-treatment	Post-treatment	z	p-value
Pre-switch	0.720 (0.325,1.015)	0.700 (0.325,1.010)	-1.061	0.288
Post-switch	0.700 (0.325,1.010)	0.580 (0.270,0.980)	-0.175	0.861

## Discussion

Aflibercept significantly reduced CT and 17 out of 20 eyes responded after switching to aflibercept in terms of IRCR. Bevacizumab and ranibizumab failed to show a significant decrease in CT and achieved neither complete resolution nor near complete resolution in IRCR evaluations. Despite those findings, no clinical implications, as reflected in BCVA, were apparent from switching injections. The characteristics shared by the eyes in the study were defect in RPE and ELM (100%), the existence of hyperreflective foci was observed in 16 eyes (80.0%) and choroidal hyperpermeability was observed in 12 out of 13 of the patients who had indocyanine green angiography examination (92.3%). 

In clinical practice, a common approach for patients with PNV who are refractory to or exhibit a suboptimal response to a single drug is to switch to a different drug. Several studies have established aflibercept's superiority to bevacizumab and ranibizumab in the treatment of PNV or PCV. Karasu et al. found patients treated with aflibercept required fewer injections and significantly decreased subfoveal CT compared to bevacizumab [9]. Corroborating our results in this study, a previous study showed that 14 PNV patients unresponsive to ranibizumab had a significant response to aflibercept. Those researchers postulated that aflibercept's wider range of targets (including placental growth factor), compared with bevacizumab and ranibizumab, and its strong binding action on the choroid, could explain this observation [10]. Most of the work highlights wide binding ability and the superior anti-VEGF action of aflibercept on choroid. Koizumi et al. were the first to suggest the link between aflibercept’s impact on CT and disease suppression in PCV [11]. Their 12-month case series showed a significant correlation between BCVA improvement and subfoveal CT reduction in PCV, but not in typical age-related macular degeneration. The authors attributed this effect to VEGF suppression, potentially via inhibition of choroidal vascular hyperpermeability and aflibercept's broader mechanism of action [11].

In PNV patients, by the inherent characteristic of the disease, the origin of the intraretinal fluid in the cysts could potentially arise from two sources: neovascularization or hyperpermeability of the choroid. In our study primary outcome showed a stronger CT reduction ability of aflibercept compared to the other anti-VEGF, which seems to be reflecting the idea of alleviating pachychoroid-driven mechanism. Moreover, not only the CT but also the size of the Haller's vessel at the same location has decreased (Figure 4A dotted rectangle). RPE atrophy and ELM defect located beneath intraretinal cysts provide a pathway of choroidal fluid entry, in which all of the cases had (Table 1 and Figure 2) choroidal vascular hyperpermeability, which is a common feature in PSD, also supports the possibility of fluid source from choroid, which was found in many eyes of the current study (Table 1). These findings, taken with the result of intraretinal cyst resolution seem to serve as supporting evidence that some portion of the intraretinal fluid is from choroid. Also, note that CT and choroidal vascularity index are reflective of disease activity in neovascular age-related macular degeneration. Thus, reduction of choroid thickness and reduction of intraretinal cyst appears to be logical.

CT in normal subjects typically falls within the range of 191 to 350 µm [4]. However, this thickness is subject to be variable influenced by several factors, including age, axial length, and the timing of measurement [12,13]. Some individuals may exhibit a thicker choroid without any discernible alterations in the RPE or the neurosensory retina, while others may have a thinner choroid accompanied by changes in the RPE and manifestation of MNV. This variability can be attributed to pathologically dilated Haller’s vessel and the attenuation of choriocapillaries above it [14]. As a result, individuals with a normal or thin choroid may still share clinical characteristics with PSD. In advanced cases, choroidal thickness may further decrease, and pachyvessels may appear to occupy the entire choroid when observed using OCT. Spaide has referred to these conditions as age-related choroidal atrophy [15]. The cases involved in this study are long-standing PNV patients who are not treatment-naïve but have a history of prior anti-VEGF therapies. Consequently, at the time of evaluation, the medial choroidal thickness values range from 181.79 µm to 274.46 µm (Table 1), which are different from what is typically considered “pachy.” Nevertheless, the presence of dilated Haller’s vessels beneath the disease focus, particularly within the CME lesion, still suggests that the pathophysiology of intraretinal fluid can be attributable to the choroidal source. 

Despite the resolution of intraretinal cysts and choroidal thickness, this study demonstrated no significant changes in BCVA at visit 2 and visit 3. A similar result was also reported in other studies that observed patients who converted from bevacizumab to another type of injection and showed anatomical improvement but no functional benefit [16,17]. The lack of correlation between IRCR and BCVA may be attributed to the extent of ellipsoid zone disruption and damage to the RPE and ELM [18]. Notably, this study included patients with prolonged treatment history with RPE atrophy and poor initial BCVA at visit 1: the initial median BCVA of the patients in this study was logMAR 0.72. Our study examined patients with varying degrees of inner and outer retina defect and observed definitive ellipsoid zone, RPE, and ELM disruption in all cases which could potentially explain the non-significant improvement in BCVA. This offers limited prospects for functional improvement, given the accompanying irreversible changes in the RPE and outer retinal layers.

Cystoid retinal degeneration, also known as a retinal pseudocyst, is characterized by empty spaces in the retina, often in the inner nuclear layer [19,20]. The cases in this study could be confused with that kind of degeneration because of similar morphology in OCT findings. However, the FA patterns, hyperfluorescence, and treatment response can demonstrate that the intraretinal fluids in this study are not simply degenerative cysts. Moreover, they are not classic CME either. Classic CME reveals a petaloid pattern in FA, but the cases in this study revealed only limited leaks and no complete CME-like petaloid pattern, although four cases did show partial petaloid patterns.

Limitations

This study has several limitations. The retrospective design of this study inherently introduces potential bias. For example, selecting the baseline visit may be controversial in terms of being “recalcitrant” because some patients experienced switching before the baseline visit. However, considering the intravitreal half-life of the drugs, the previous switching would not impact the investigation and still provide meaningful data. However, a follow-up study on this topic should address this issue. Further limitation pertains to the sample size. Only a small number of cases were treated with aflibercept and bevacizumab, and the number of patients treated with ranibizumab was extremely small. This may influence the statistical analysis. Moreover, the treatment protocols were not controlled and the number and type of previous treatments vary, which may serve as a confounding factor. Future studies involving more participants with a control group would strengthen this research topic.

## Conclusions

This study aimed to define the clinical characteristics and treatment effects of different intravitreal medications in patients with pachychoroid neovasculopathy (PNV) accompanied by recalcitrant intraretinal cysts. The findings demonstrated that aflibercept was more effective than bevacizumab or ranibizumab in reducing choroidal thickness (CT) and resolving intraretinal cysts that were previously unresponsive to other anti-vascular endothelial growth factor therapies. The significant reduction in CT and the high rate of intraretinal cyst resolution with aflibercept highlight its superior efficacy in addressing the pathophysiological features of PNV, particularly when driven by choroidal hyperpermeability rather than purely neovascular activity. Despite these anatomical improvements, no significant functional changes were observed, which appears to be due to chronic damage on the outer retinal layers. These results underscore the importance of considering both the underlying mechanisms of fluid accumulation and the persistence of intraretinal cysts when selecting treatment options for PNV.
